# Are pharmacological randomised controlled clinical trials relevant to real-life asthma populations? A protocol for an UNLOCK study from the IPCRG

**DOI:** 10.1038/npjpcrm.2016.16

**Published:** 2016-04-14

**Authors:** Karin Lisspers, Pedro Teixeira, Coert Blom, Janwillem Kocks, Björn Ställberg, David Price, Niels Chavannes

**Affiliations:** 1 Department of Public Health and Caring Science, Family Medicine and Preventive Medicine, Uppsala University, Uppsala, Sweden; 2 ICVS/3B's - PT Government Associate Laboratory, Life and Health Sciences Research Institute (ICVS), School of Health Sciences, University of Minho, Braga, Portugal; 3 Department of Primary and Community Care, Radboud University Nijmegen, Nijmegen, The Netherlands; 4 Department of General Practice, University of Groningen, University Medical Center Groningen, Groningen, The Netherlands; 5 GRIAC Research Institute Groningen, University of Groningen, University Medical Center Groningen, Groningen, The Netherlands; 6 Academic Primary Care, Division of Applied Health Sciences, University of Aberdeen, Aberdeen, UK; 7 Public Health and Primary Care, Leiden University Medical Center, Leiden, The Netherlands

## Introduction

Asthma has a high prevalence worldwide with a high incidence in primary care settings in many countries.^1^ It is by definition a variable disease with a broad spectrum of clinical phenotypes, in which management and treatment can be difficult.^2–8^ The aim of asthma treatment is optimal control of the disease, which according to Global Initiative for Asthma (GINA) guidelines implies both symptom control and prevention of exacerbations.^1^ Despite several treatment options, studies show that about half of the patients have poor asthma control.^2,3^ When asthma is not controlled, it decreases the quality of life, increases the risk of exacerbations and premature death and is a high cost for the society.^2,3^

There are concerns that current asthma treatment is based on research with subjects who are not representative of the patients seen in clinical practice.^9^ Guidelines for the management of asthma are usually developed on the basis of the available empirical evidence, and particular emphasis is placed on the conclusions of randomised controlled clinical trials (RCTs) and meta-analyses of RCTs that have been placed at the top of the evidence hierarchy.^10^ However, most RCTs have restricted inclusion criteria to obtain a high level of internal validity and usually recruit patients from secondary health-care settings. The external validity of RCTs, which provides evidence for major clinical guidelines, may be questioned if they are not representative of real-life populations in primary care.^11–15^ In the 2007 study by Travers *et al*, only 4% (range 0–36%) of participants with asthma in a population-based survey met the eligibility criteria to be included in 17 major RCTs cited in the GINA guidelines.^14^ The same study found that only 6% (range 0–43%) of participants receiving asthma treatment also met the eligibility criteria. The proportion of patients with asthma from primary care settings that would be eligible for the major RCTs is unknown. If the proportion is similar to that found in the study by Travis *et al.*, then the generalisability of the conclusions and the clinical relevance of major RCTs may be questionable, and they have not been improved in the past decade. There is a need to explore the external validity of pharmacological RCTs in primary care populations. The UNLOCK (Uncovering and Noting Long-Term Outcomes in COPD and asthma to enhance knowledge) project^15^ of the International Primary Care Respiratory Group (IPCRG) covers a broad primary care population that may help clarify the external validity of these RCTs.

## Aims and objectives

The aim of this study is to determine whether the inclusion criteria for patients with asthma in the major RTCs supporting GINA guidelines are representative of real-life primary care populations with asthma. The study will address the following research questions:

1. What proportion of patients in primary care would be eligible for the major RCTs assessing patients at treatment step two heading for step three?
2. What are the clinical characteristics of these patients when compared with participants in major RCT studies?

## Discussion

Asthma is a highly prevalent disease worldwide with variability and a large range of phenotypes.^1–8^ The management and treatment of patients with asthma in primary care is a challenge, and many patients still suffer from poor asthma control.^2^ The major international clinical guidelines for the prevention and management of asthma are influenced by the empirical evidence and conclusions of RCTs.^9–14^ However, RCTs tend to be highly selective in terms of inclusion and exclusion criteria for people with asthma and may under-represent primary care populations with asthma. Smokers, for example, are usually excluded from registration RCTs assessing inhaled corticosteroids (ICS), as smoking is known to reduce the efficacy of ICS.^16^ Other common exclusion criteria are pregnancy, lactation, elderly patients and other chronic diseases such as ischaemic heart disease. A common inclusion criterion is a bronchodilator FEV_1_ reversibility of 12 or 15%, which is not a frequent clinical sign in real-life asthma patients, and in the Travers study, 71 (or 76%) of participants with asthma were excluded.^14^

RCTs are important in establishing the efficacy and short-term safety of new therapies, but there are limitations in evaluation of effectiveness of the therapies in the real world.^17^ The assessment of the external validity of asthma RCTs is therefore needed to better understand the clinical relevance of their conclusions. There should be discussion on the need for effective studies, pragmatic trials and observational studies, as a complement to RCTs, to evaluate treatment outcomes in real-life clinical settings including the whole asthma population. The main weakness of these studies might be a lower internal validity, which needs to be addressed in the design of the studies. When developing guideline recommendations in the future, data from both efficacy and effectiveness studies should be evaluated with the strengths and weaknesses given.^18^

This study will provide estimates of the proportion of real-life primary care populations that are eligible for RCTs. It will also present descriptive characteristics of primary care patients compared with patients included in RCTs. This will present relevant information for the adequacy and use of GINA and other major guidelines in primary health-care settings.^16–20^ Previous findings suggest that the level of representation of the population with asthma^14^ and chronic obstructive pulmonary disease (COPD)^13^ in RCTs may be lower than desirable. This study can inform not only the development of future clinical practice guidelines^17–20^ but also the way asthma clinical trials are being designed and implemented.^10^ The study will tell us more about the patients who are not eligible for these major RCTs, and it may also provide new insights into why the control of asthma in many patients does not appear to improve in spite of the implementation of the treatment strategies that are recommended in guidelines.

## Materials and methods

### Study design

The study will use an observational design for comparing baseline characteristics of asthma patients in primary care databases with the inclusion criteria in the pharmacological RCTs that support major international guidelines (i.e., GINA).

### Data collection and inclusion criteria

Data collection will involve two steps:

1. Step one will identify the major relevant RCTs used in GINA guidelines.
2. Step two will identify the primary care data sets that contain the required variables to assess eligibility for participation in the major RCTs.

In step one, a Systematic Review focusing the search on selected asthma international guidelines (i.e., GINA) will be conducted in order to identify all studies that fit the following criteria:

- Randomised controlled clinical trial
- Pharmacological treatment at GINA management step 2 heading for step 3
- RCT sample size *n*>400
- Other criteria (e.g., mainly adult population)

**Figure 1:**
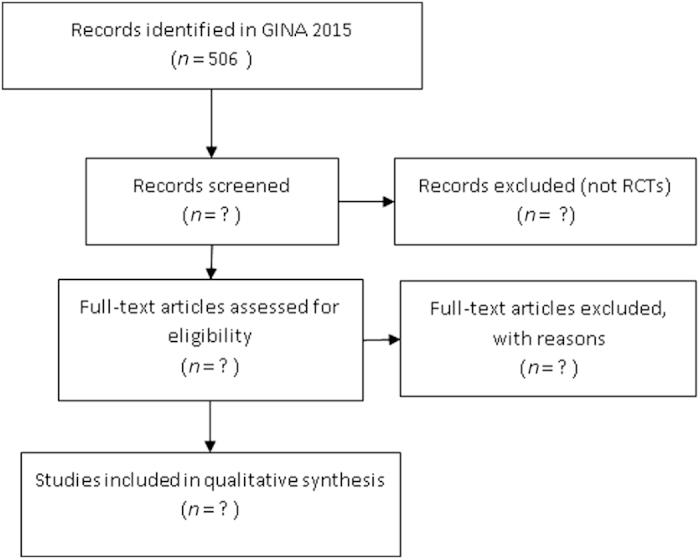
Flowchart of RCT identification process.

The systematic review will follow the PRISMA^21^ statement guidelines and follow the steps identified in Figure 1.

In step two, members of the IPCRG UNLOCK group will be invited to participate in the study with primary care data sets that include the variables listed in Table 1.

Participants identified in the data sets will remain anonymous. Patient confidentiality will be assured in the collection and merging of the data sets.

### Data analysis

Data will be analysed according the defined criteria for the identification of RCTs. Statistical analysis of the data sets will provide summary descriptive statistics, means, measures of dispersion and proportions. The statistical analysis will focus on group classification values, and no individual statistical values will be computed or revealed.

### Ethical approval

All included data sets will require approval by local primary care research ethics committees.

## Figures and Tables

**Table 1:** List of variables for data set inclusion in the study

*Variables*	*Required*	*Optional*
Age (>12 years)	√	
Gender	√	
Medication: ICS, LABA, SABA, combination, Montelukast—(last 3 months) fixed combination (ICS/LABA)	√	
*One of:*		
Asthma control (GINA or ACQ or ACT)	√	
QoL (miniAQLQ)		
		
Smoking history: (never–former–current)		√
		
*Latest lung function assessment*		
FEV_1_ % of predicted		√
FEV_1_ reversibilty		
FEV_1_/FVC (VC) ratio		
		
Allergy (pollen, pets, house dust mite)		√
Allergic rhinitis		√
Comorbidities heart disease (i.e., heart failure or ischaemic heart disease)		√
Asthma control (i.e., according to GINA: last week: daytime symptoms, night symptoms rescue medication and exacerbations history, courses of oral steroids history or ACQ or ACT)		√
QoL (i.e., miniAQLQ)		

Abbreviations: ACQ, asthma control ouestionnaire; ACT, asthma control test; ICS, inhaled corticosteroids; LABA, long-acting β-agonists; miniAQLQ, mini quality of life questionnaire; QoL, quality of life; SABA, short-acting β-agonists.
